# Spontaneous void formation in antimony alloy anodes

**DOI:** 10.1038/s42004-020-00350-9

**Published:** 2020-08-07

**Authors:** Teresa S. Ortner

**Affiliations:** Communications Chemistry, https://nature.com/commschem

## Abstract

Lithium-ion batteries suffer from stability issues. Now, spontaneously formed, stable voids in antimony alloy anodes offer a simple remedy.

Instability is a problem for lithium-ion batteries, largely owing to changes in electrode structure and volume during cycling. Efforts have been made to improve stability, for example by developing hollow^1^ and yolk-shell anode structures^2^ that can accommodate volume changes in their inner voids, but their large-scale syntheses are challenging and expensive. Now, Matthew T. McDowell from Georgia Institute of Technology and colleagues from the US and Switzerland show how 15-nm-sized antimony nanocrystals spontaneously form stable and uniform voids when lithium is removed from the anode during cycling. They report an efficient and stable lithium-ion battery electrode without the need for multi-step processing (https://www.nature.com/articles/s41565-020-0690-9)^3^.

“Investigating reaction mechanisms in small, monodisperse metal nanocrystals was of interest at the outset of this project because of the possibility of size-dependent and novel behavior,” says McDowell, “but the spontaneous voiding mechanism that we observed in the experiments was not anticipated.” Studying this unexpected behavior, the team found that the formation of voids is driven by a resilient, native antimony oxide layer. In contrast to the generally held view that a native oxide is detrimental, the group shows that it is, instead, a critical feature in this material. The oxide layer allows for expansion of the particle when lithium initially enters, but mechanically prevents shrinking upon delithiation. An inner antimony coating of the shell is retained upon delithiation, and upon repeated insertion of lithium, the Li-Sb nanoparticle is recovered, keeping its expanded size and the thickened oxide layer (Fig. 1). As a result, volume contractions and changes in surface dimensions, which bear on anode stability and result in low cyclability, are minimal. An average Coulombic efficiency of 99.4% is achieved for the 30th–100th cycles, which is comparatively high for a nanoparticle-based electrode.

**Fig. 1:**
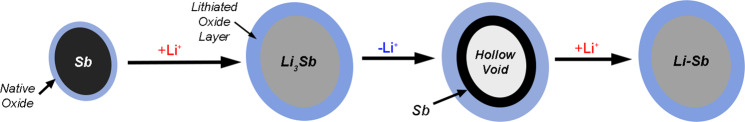
Schematic of the anode cycling process. Antimony nanoparticles covered in a native antimony oxide layer (light blue) expand upon initial lithiation. A hollow void, lined by an inner antimony layer (black), is formed upon delithiation. The expanded size is retained when lithium reenters. Reprinted by permission from Springer Nature: Nat. Nanotechnol. 15, 475–481, copyright 2020.

While monodisperse antimony nanocrystals with diameters of 10–20 nm are known to display superior cyclability to their micron-sized counterparts^4^, the role of nanoparticle size remained unclear. Based on their experimental results, McDowell and team developed a chemomechanical model to explain the hollowing behavior and its size-dependence. In agreement with in situ transmission electron microscopy experiments, the model shows that smaller particles (<~30 nm) should form hollow voids during delithiation, while larger particles should instead shrink while mechanically “buckling” the oxide shell. This behavior is driven by the energetic balance between forming new void surfaces and mechanically deforming the shell.

The growth model suggests that voiding transformations don’t have to be limited to Li-ion systems. “Antimony is one of the relatively few materials that can react reversibly with lithium, sodium, and potassium,” concludes McDowell, “The new understanding and concepts in our paper could therefore also benefit the development of antimony-based anodes for these emerging battery systems.”
